# Platelets of mice heterozygous for neurobeachin, a candidate gene for autism spectrum disorder, display protein changes related to aberrant protein kinase A activity

**DOI:** 10.1186/2040-2392-4-43

**Published:** 2013-11-04

**Authors:** Kim Nuytens, Krizia Tuand, Michela Di Michele, Kurt Boonen, Etienne Waelkens, Kathleen Freson, John WM Creemers

**Affiliations:** 1 Department of Human Genetics, Laboratory for Biochemical Neuroendocrinology, KU Leuven, 3000 Leuven, Belgium; 2 Leuven Autism Research Consortium (LAuRes), KU Leuven, 3000 Leuven, Belgium; 3 Department of Cardiovascular Sciences, Center for Molecular and Vascular Biology, KU Leuven, 3000 Leuven, Belgium; 4 Department of Cellular and Molecular Medicine, Laboratory of Protein Phosphorylation and Proteomics, KU Leuven, 3000 Leuven, Belgium

**Keywords:** Autism spectrum disorder, AKAP, Calpain, Candidate gene, Neurobeachin, Platelets, Serotonin, Talin-1

## Abstract

**Background:**

*Neurobeachin* (*NBEA*) has been identified as a candidate gene for autism spectrum disorders (ASD) in several unrelated patients with alterations in the *NBEA* gene. The exact function of NBEA, a multidomain scaffolding protein, is currently unknown. It contains an A-kinase anchoring protein (AKAP) domain which binds the regulatory subunit of protein kinase A (PKA) thereby confining its activity to specific subcellular regions. NBEA has been implicated in post-Golgi membrane trafficking and in regulated secretion. The mechanism of regulated secretion is largely conserved between neurons and platelets, and the morphology of platelet dense granules was found to be abnormal in several ASD patients, including one with NBEA haploinsufficiency. Platelet dense granules are secreted upon vascular injury when platelets are exposed to for instance collagen. Dense granules contain serotonin, ATP and ADP, which are necessary for platelet plug formation and vascular contraction.

**Methods:**

To further investigate possible roles for NBEA in secretion or dense granule morphology, platelets from Nbea^+/-^ mice were analyzed morphometrically, functionally and biochemically. A differential proteomics and peptidomics screen was performed between Nbea^+/-^ and Nbea^+/+^ mice, in which altered Talin-1 cleavage was further investigated and validated in brain samples. Finally, the phosphorylation pattern of PKA substrates was analyzed.

**Results:**

Platelet dense granules of Nbea^+/-^ mice had a reduced surface area and abnormal dense-core halo, but normal serotonin-content. Nbea haploinsufficiency did not affect platelet aggregation and ATP secretion after collagen stimulation, although the platelet shape change was more pronounced. Furthermore, peptidomics revealed that Nbea^+/-^ platelets contain significantly reduced levels of several actin-interacting peptides. Decreased levels were detected of the actin-binding head and rod domain of Talin-1, which are cleavage products of Calpain-2. This is most likely due to increased PKA-mediated phosphorylation of Calpain-2, which renders the enzyme less active. Analysis of other PKA substrates revealed both increased and reduced phosphorylation.

**Conclusion:**

Our results show the pleiotropic effects of alterations in PKA activity due to Nbea haploinsufficiency, highlighting the important function of the AKAP domain in Nbea in regulating and confining PKA activity. Furthermore, these results suggest a role for Nbea in remodeling the actin cytoskeleton of platelets.

## Background

The genetic architecture of autism spectrum disorders (ASDs) is highly heterogeneous and to date more than 100 genes have been reported to be deleted, duplicated, mutated or disrupted by a translocation breakpoint in ASD patients [1,2]. One of these candidate genes, *Neurobeachin* (*NBEA*) [MIM: 604889] was identified in a patient with a *de novo* balanced chromosomal translocation t(5;13)(q12.1;q13.2) with one breakpoint in intron 2 of *NBEA* resulting in a NBEA haploinsufficient status [3]. Additionally, four unrelated ASD patients with a monoallelic deletion of *NBEA* were reported [4-7], and three novel CNVs were detected within the *NBEA* gene in three unrelated individuals diagnosed with ASD [8-10]. Moreover, a single nucleotide polymorphism (SNP) in intron 38 of *NBEA* has been associated with ASD [11]. The *NBEA* gene contains a low-frequency common fragile site (*FRA13*) linked to ASD and is located in a 19 cM region identified as a candidate region for ASD by a linkage study (MMLS/het score of 2.3 between markers D13S217 at 17.21 cM and D13S1229 at 21.51 cM) [12-14].

NBEA, a brain-enriched multidomain scaffolding protein, is located at the tubulovesicular endomembranes near the *trans*-Golgi network [15,16]. The N-terminal region contains a Concanavalin A-like lectin domain flanked by armadillo repeats suggested to play a role in intracellular trafficking [17,18]. Distal from these regions, an A-kinase anchoring protein (AKAP) domain is present, recruiting cAMP-dependent protein kinase A (PKA) by high-affinity binding to its regulatory RIIα subunit [16]. The C-terminal part of NBEA possesses a pleckstrin homology - beige and Chediak-Higashi (BEACH) - WD40 domain module which is thought to be implicated in vesicle trafficking [16,19]. NBEA and eight other human proteins contain the highly conserved BEACH domain, and thus belong to the family of BEACH proteins [20].

Although the exact function of NBEA is currently unknown, a complete loss of Nbea in mice leads to a perinatal lethal phenotype due to a complete block of evoked neuromuscular transmission [21]. By studying neuronal cultures derived from E18 Nbea^-/-^ mice, a role emerges for Nbea in trafficking important cargo to pre- and postsynaptic compartments, as these cultures have shown abnormal large clusters of actin in the soma, dendritic shafts and axons, and a reduced level of neurotransmitter receptors has been detected at the surface of the postsynaptic membrane [22,23]. Moreover, knockdown of Nbea in a neuroendocrine cell line (βTC3 cells) leads to enhanced secretion of dense-core secretory granules, the neuroendocrine counterpart of large dense-core vesicles (LDCVs) in neurons, making Nbea a negative regulator of the regulated secretion [24].

Blood platelets are the first players to be activated upon vascular injury. They are essential for initiating platelet plug formation and do so by secreting the content of their secretory granules. Similar to neurons, platelets contain two types of secretory granules, namely the alpha and dense granules, corresponding to the small synaptic vesicles (SVs) and LDCVs in neurons, respectively [25]. Blood platelet alpha granules have a heterogeneous cargo of polypeptides ranging from adhesion molecules to growth factors, whereas dense granules contain the small molecules ATP, ADP and serotonin, necessary for vasoconstriction. Due to the similar regulation of granule formation and transport, platelets were put forward as a model system to study the biology of granule formation, trafficking and secretion in neurons [26,27]. Preliminary *in vivo* analysis unveiled an abnormal morphology of the dense granules in platelets of our reported patient with a chromosomal translocation in *NBEA*[24]. Moreover, similar platelet granule abnormalities were observed in ASD patients with chromosomal rearrangements in *Amisyn* or *SCAMP5*, and in an ASD patient with a deletion including *SHANK3*[24]. Interestingly, mutations in LYST and NBEA-like 2 (NBEAL2), two other BEACH proteins, are described in patients with Chediak-Higashi and Gray platelet syndrome and result in abnormal to absent platelet dense and alpha granules, respectively [28,29].

As platelets can easily be obtained from patients, further insights into platelet abnormalities might lead to the identification of biomarkers associated with ASD. We have characterized platelets from mice heterozygous for Nbea to substantiate the causality of NBEA haploinsufficiency for the abnormal platelet phenotype. The ultrastructure of the dense granules of murine platelets was analyzed and platelet function was investigated. Moreover, serotonin levels were determined in both serum and platelets, as hyperserotonemia is the only biochemical anomaly reported in approximately 30% of ASD patients. Serotonin is a hormone and monoamine neurotransmitter that can induce vasoconstriction and is implicated in neuron outgrowth, maturation, function and plasticity. It is synthesized in serotonergic neurons of the central nervous system and in the intestine, and more than 99% of whole blood serotonin is stored in blood platelets [30]. To assess whether Nbea haploinsufficiency affects the protein and peptide content of platelets, a full proteomic and peptidomic analysis was performed and results were further validated in platelets and in total brain.

## Methods

All experiments were approved by the ethical research committee of KU Leuven in accordance with the declaration of Helsinki (project number P182/2011).

### Animals

The GH240B transgenic line described in Su *et al.*[21] was backcrossed for at least 10 generations with C57BL/6JRj mice (Janvier). Peripheral blood samples were obtained from adult (12-week-old) female mice.

Brains were dissected from 12-week-old mice and immediately put at -80°C. Tissue was homogenized in sucrose buffer (3.18 mM sucrose; 4 mM Tris–HCl pH 7.4) containing a protease and phosphatase inhibitor cocktail and a complete protease inhibitor cocktail (both from Roche Applied Science, Penzberg, Germany).

### Platelet function analysis and platelet counts

Murine blood was anticoagulated with 3.2% trisodium citrate (9:1) and mean platelet volume (MPV) and platelet count were determined on an automated cell counter (Cell-Dyn 1300 Abbott laboratories, Abbott Park, IL, USA). Platelet-rich plasma (PRP) was obtained after centrifugation at 3,000 rpm for 30 sec followed by 800 rpm for 5 minutes. The platelet count was adjusted to 250,000 platelets/μl with autologous plasma. Platelet aggregation and secretion were performed as described after stimulation with Horm collagen (5 μg/ml) [31,32]. Platelet secretion was determined by measuring the release of ATP using luciferin/luciferase reagent (Kordia, Leiden, The Netherlands). Electron microscopy analysis of murine platelets was performed as previously reported [33]. Additional ultra-thin sections of 50 to 70 nm were cut, stained with uranyl acetate and lead citrate, and examined at 80 kV using a JEM1400 transmission electron microscope (JEOL, Tokyo, Japan). Micrographs were acquired on an SIS Quemesa camera (Olympus, Münster, Germany). The number of dense granules per platelet and dense granule dimension and morphology were further assessed with the ImageJ imaging system (National Institutes of Health, Bethesda, MD, USA) (n = 200 platelets/genotype). Dense granules were classified as different types: type 1, solid core occupying more than 50% of the granule; type 2, solid core occupying less than 50% of the granule; type 3, fragmented core; or type 4, empty granule/no visible core [34,35].

### Determination of platelet size and distribution by flow cytometry

Integrin αIIbβ3 expression was measured in whole blood by incubation with fluorescein isothiocyanate (FITC)-conjugated anti-CD41/61 monoclonal antibody (BD Biosciences, Bergen County, NJ, USA) for 15 minutes. Forward and side scatter and percentage platelets to total cell number were analyzed using FACSDiva version 6.1.2 software (BD Biosciences) on a FACSCalibur flow cytometer (BD Biosciences).

### Platelet isolation for protein analysis

Peripheral blood samples were obtained from the retro-orbital sinus (anticoagulated with ACD pH6.5 (7 mM citric acid; 93 mM sodium citrate; 140 mM dextrose); 9:1). PRP was obtained as described above. Platelets were obtained by PRP centrifugation at 2,300 rpm for 10 minutes and washed twice with ACD pH6.5. For proteomic purposes, PRP of littermates with the same genotype was pooled to yield sufficient protein contents to prepare the platelet pellets.

### Determination of serotonin levels in platelets and serum

Serum was obtained from blood coagulated for 30 minutes at 37°C in glass cuvettes followed by centrifugation at 2,300 rpm for 10 minutes. Serotonin content of platelets, isolated as mentioned above, and serum was calculated using the serotonin research ELISA (Labor Diagnostika Nord, Nordhorn, Germany) according to the protocol of the manufacturer (n = 8 mice/genotype).

### Two dimensional-differential gel electrophoresis (2D-DiGE)

Platelet pellets (n = 4 samples/genotype) were lysed in DiGE lysis buffer containing 7 M urea, 2 M thiourea, 4% CHAPS and 30 mM Tris pH 8.5 and a complete protease inhibitor cocktail (Roche Applied Science, Penzberg, Germany). The samples were purified with the 2D Clean-Up Kit (GE Healthcare, Buckinghamshire, UK) and the concentration was determined using the 2D Quant Kit (GE Healthcare) according to the manufacturer’s guidelines. Proteins were labeled with carbocyanine (Cy) dyes as previously described [31]. Briefly, 50 μg of each sample was labeled with 200 pmol of Cy3 or Cy5. To avoid possible bias due to labeling efficiency, two samples of each genotype were labeled with Cy3 and the other two with Cy5. The internal standard consisting of a pool of all samples was labeled with Cy2 allowing a quantitative comparison for a protein of two samples resolved on the same gel (ratio Cy3/Cy2 and Cy5/Cy2) and a quantitative comparison of multiple gels. Mixtures of Cy3-, Cy5- and Cy2-labeled samples were diluted 1:1 with lysis buffer containing 0.5% IPG buffer (pH 4 to 7) and 1.3% dithiothreitol (DTT) and applied by cup loading on rehydrated IPG strips (pH 4 to 7, 18 cm). The first dimension was carried out in an IPGphor system (GE Healthcare) with the following conditions: 1 h 30 minutes at 150 V, 2 h at 500 V, 5 h at 1,000 V, 3 h at 8,000 V in gradient and 5 h at 8,000 V. IPG strips were subsequently incubated in equilibration buffer (6 M urea, 30% glycerol, 2% SDS and 50 mM Tris pH 8.5) supplemented with 65 mM DTT for 20 minutes and followed by incubation in equilibration buffer supplemented with 200 mM iodoacetamide and 0.02% bromophenol blue for 20 minutes. The second dimension was performed on 11% polyacrylamide gels on the Hoefer DALT vertical system (GE Healthcare). Visualization and analysis of the images as well as the identification of differentially expressed proteins were executed as described previously [31].

### Immunoblot analysis

Platelet pellets were resolved in lysis buffer (1% Igepal; 0,015% DTT; 1 mM ethylene diamine tetraacetic acid (EDTA) in PBS supplemented with a complete protease inhibitor cocktail) (Roche Applied Science). Protein concentration of the platelet and brain lysates was quantified with a bicinchoninic acid (BCA) protein assay (Thermo Scientific, Rockford, IL, USA). Depending on the molecular weight of the protein of interest, 25 μg of platelet or brain lysates was loaded on a 10% Bis-Tris gel (Bio-Rad, Hercules, CA, USA) or 3 to 8% Tris-Acetate gel (Life technologies, Paisley, UK). Proteins were transferred to Protan Nitrocellulose membrane (Schleicher & Schuell, Dassel, Germany) and incubated with antibodies against Munc13-4 (Santa Cruz Biotechnology, Santa Cruz, CA, USA; 1/1,000), Rab27b (homemade antibody, 1/1,000), Calmodulin (kindly provided by Professor H Desmedt, KU Leuven; 1/1,000), Talin-1 (Cell Signaling Technology, Danvers, MA, USA; 1/500), Calpain-2 large subunit (Cell Signaling Technology; 1/1000), Calpain-4 regulatory subunit (Santa Cruz Biotechnology; 1/1,000), phospho-(Ser/Thr) PKA substrate antibody (Cell Signaling Technology; 1/1,000), and actin (Sigma-Aldrich, St Louis, MO, USA; 1/5,000), used for normalization. Equal amounts of actin protein expression were verified after incubation with an anti-glyceraldehyde-3-phosphate dehydrogenase (GAPDH) antibody (Abcam, Cambridge, UK; 1/15,000). Afterwards, membranes were incubated with horseradish peroxidase (HRP)-conjugated secondary antibody (1/2,000; DAKO, Glostrup, Denmark) and proteins were visualized with western blotting ECL detection reagent. Quantification was performed using the Kodak Imager software (Kodak, Rochester, NY, USA).

### Immunoprecipitation

Per sample, 20 μl of 50% bead slurry of protein A agarose beads (GE Healthcare) was used and washed twice with PBS before use. All incubations were performed at 4°C on a mechanical rotator. Prior to immunoprecipitation, pre-clearing of the platelet or brain sample was performed as follows. Protein A agarose beads were incubated with rabbit serum (ImmuniBioScience, Mukiltoe, WA, USA) for 1 h after which 60 μg of platelet or brain sample was added for 1 h. The immunoprecipitation was subsequently performed with the pre-cleared supernatant by adding phospho-(Ser/Thr) PKA substrate antibody (Cell signaling technology; 1/100) for overnight incubation, followed by an additional 2 h incubation with protein A agarose beads. Beads were washed five times with PBS and proteins were harvested by resuspension of the beads in sample buffer (50 mM Tris–HCl pH 7; 10% glycerol; 2% SDS; bromophenol blue) compatible with immunoblot analysis.

### Peptidomics

Platelets were isolated as described above with modification of the final wash buffer, which was now replaced by PBS. The procedure for processing the platelet pellets (n = 5 mice/genotype) for mass spectrometry analysis was performed as reported [36]. DeCyder MS 2.0 (GE Healthcare) is a differential analysis software tool that also allows for easy visualization of liquid chromatography mass spectrometry (LC-MS) runs by creating artificial two-dimensional maps with the *m/z* values and retention times in the first and second dimension, respectively. Time alignment, intensity normalization and statistics were performed using this software. Peptides were identified in additional LC-MS/MS runs of the pooled samples as reported in [36] using LC quadrupole time-of-flight (Q-TOF) MS.

### Statistical analysis

Data are presented as mean ± standard error of the mean (SEM). Significance of differences was analyzed using (where appropriate) the two-tailed *t*-test, *t*-test for single means, Mann–Whitney *U*-test (MWU) or Pearson Chi-square test using Statistica version 9.0 (StatSoft Inc., Tulsa, OK, USA). All statistical tests were performed with 0.05 as the α-level of significance (^*^P <0.05, ^**^P <0.01 and ^***^P <0.001).

## Results

### Nbea^+/-^ mice have normal platelet counts and MPV

Heterozygous Nbea mice had a similar platelet count (n = 10/genotype; two-tailed *t*-test, *P* = 0.20 (Table 1) and MPV (n = 10/genotype; two-tailed *t*-test, *P* = 0.58) (Table 1) compared to wild-type mice. The normal platelet size, distribution and count was confirmed by means of flow cytometry analysis, as no differences could be detected in forward and side scatter or in percentage CD41/61-positive platelets relative to the total blood cell-number (n = 10/genotype; two-tailed *t*-test, *P* = 0.83, *P* = 0.34 and *P* = 0.10, respectively) (Table 1).

**Table 1:** Morphological and functional characteristics of blood platelets haploinsufficient for Neurobeachin

		**Nbea**^**+/+**^	**Nbea**^**+/-**^	***P***
Platelet count (10^3^/μl)		943.6 ± 41.9	864.9 ± 42.2	0.20
Mean platelet volume (fL)		6.52 ± 0.11	6.61 ± 0.12	0.58
Flow cytometry				
	Forward scatter (AU)	13,683.3 ± 667.3	13,853.8 ± 255.9	0.83
	Side scatter (AU)	1,038.5 ± 21.5	1,012.4 ± 13.3	0.34
	Platelets/cell total (%)	9.88 ± 0.53	8.46 ± 0.57	0.10
Dense granules				
	Number/platelet	0.445 ± 0.028	0.441 ± 0.028	0.94
	Surface area (nm^2^)	18,063.1 ± 696.2	14,331.3 ± 649.7	<0.001
	Dense-core area (nm^2^)	2,375.4 ± 251.2	1,706.4 ± 183.0	0.52
	Halo area (nm^2^)	15,687.7 ± 654.1	12,624.9 ± 607.4	0.001
Dense granule classification				
	Type 1 (%)	13.4	10.6	0.73
	Type 2 (%)	26.7	29.3	
	Type 3 (%)	1.4	1.9	
	Type 4 (%)	58.5	58.2	
Serotonin content				
	Serum (ng/μl)	1.99 ± 0.25	2.21 ± 0.14	0.45
	Platelets (μg/10^9^ PLTs)	7.59 ± 1.62	6.84 ± 1.61	0.75
Aggregation (amplitude %)				
	Collagen (5 μg/ml)	54.4 ± 5.7	52.8 ± 3.8	0.82
ATP secretion (μM)				
	Collagen (5 μg/ml)	1.58 ± 0.41	1.71 ± 0.67	0.87

Platelet count and size were investigated in 10 mice/genotype. Dense granule morphology (MWU test) and classification was analyzed in 200 platelets/genotype. Dense granule classification was based on the study by Weiss *et al.*[35] and the total amount of dense granules counted per genotype was set at 100% yielding the above-mentioned separate percentage per dense granules type. The distribution of the dense granules into the classification types was compared between Nbea^+/-^ and Nbea^+/+^ mice using the Pearson Chi-square test. Serotonin content was measured in eight mice/genotype. Platelet function was assessed in four mice/genotype. ATP secretion and aggregation were measured for 5 minutes after collagen stimulation. Unless stated otherwise, parameters were compared between genotypes using the two-tailed *t*-test. Data are represented as mean ± standard error of the mean. AU, arbitrary units; PLT, platelet.

### Abnormal dense granules in platelets of Nbea^+/-^ mice

**Figure 1:**
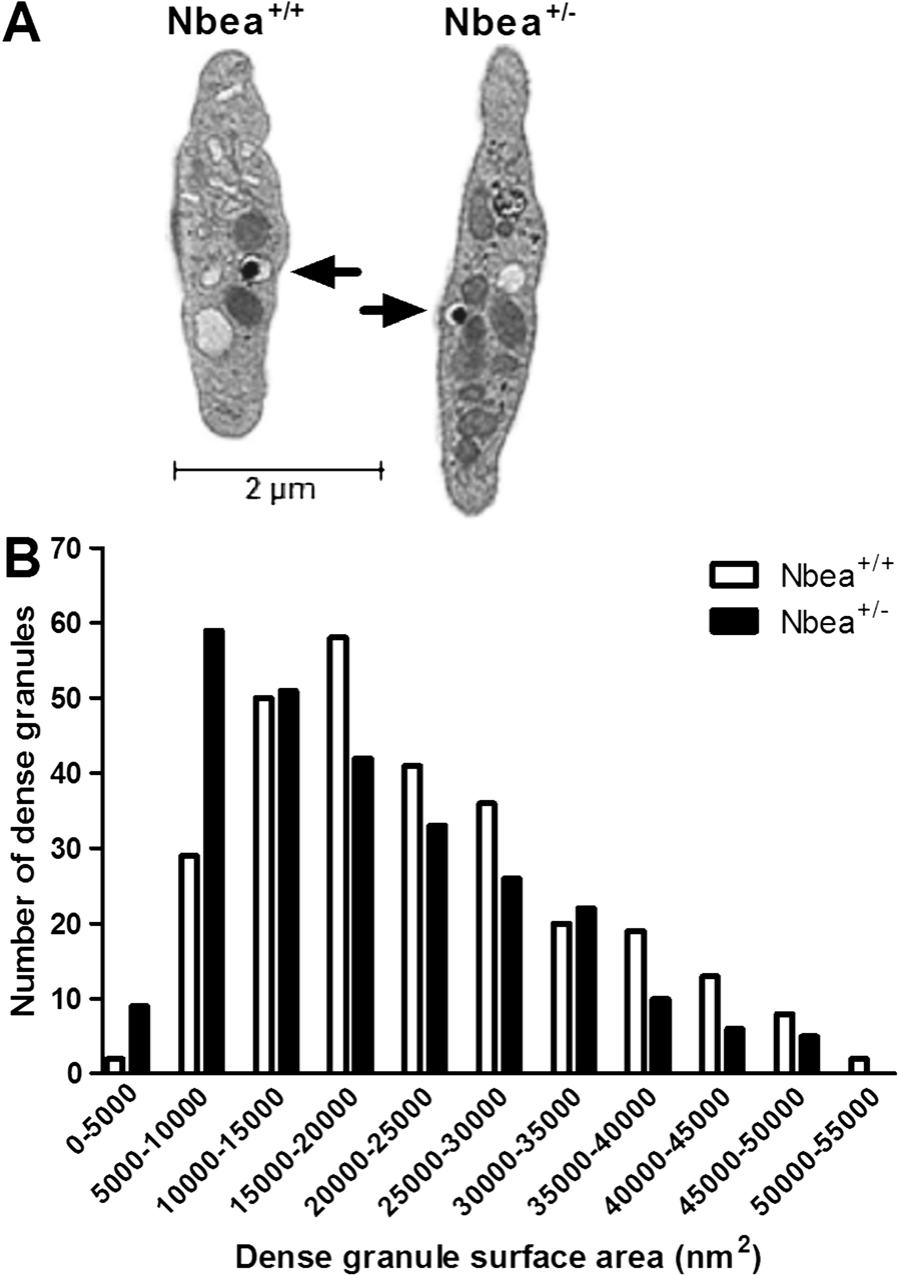
**Reduced total surface area of dense granules due to a decrease in surface area of the halo in platelets of Nbea**^**+/-**^**mice. (A)** Electron microscopic view of a blood platelet of Nbea^+/+^ and Nbea^+/-^ mice. Dense granules are indicated with black arrows. **(B)** Distribution of dense granule number according to their surface area. Dense granules of platelets of Nbea^+/-^ mice were significantly smaller than dense granules of platelets of Nbea^+/+^ mice (n = 200 platelets/genotype).

The ultrastructure of the platelets of Nbea^+/-^ mice (n = 200 platelets/genotype) was assessed to detect previously described alterations in dense granule cores as described for a patient haploinsufficient for NBEA [24] (Figure 1A). The number of dense granules per platelet was similar for Nbea^+/-^ and Nbea^+/+^ mice (MWU, *P* = 0.94) (Table 1). However, the total surface area within the limiting membrane of the dense granules of platelets of Nbea^+/-^ mice was significantly reduced (MWU, *P* <0.001) (Table 1, Figure 1), due to a reduced surface area of the halo surrounding the dense-core (MWU, *P* = 0.001) (Table 1). Based on the appearance of the dense-core, dense granules were subdivided into 4 different types according to Weiss *et al*. [35]. No significant difference between genotypes was detected in the presence of the four types of granules in platelets (Pearson Chi-square test, *P* = 0.73) (Table 1).

### Normal serotonin levels in heterozygous Nbea mice

Serotonin secreted from enterochromaffin cells enters the blood stream where it is actively taken up by platelets and stored in dense granules [37]. Platelet and serum serotonin levels were measured, but no significant difference could be detected between Nbea^+/+^ mice and Nbea^+/-^ mice (n = 8 mice/genotype; two-tailed *t*-test, *P* = 0.75 and *P* = 0.45, respectively) (Table 1).

### Subtle changes in platelet function in Nbea^+/-^ mice

Platelet function was measured by the ATP secretion and aggregation test. In line with measurements of comparable serotonin levels, dense granules from wild-type and heterozygous Nbea mice secrete similar levels of ATP upon collagen stimulation (n = 4 mice/genotype; two-tailed *t*-test, *P* = 0.87) (Table 1).

**Figure 2:**
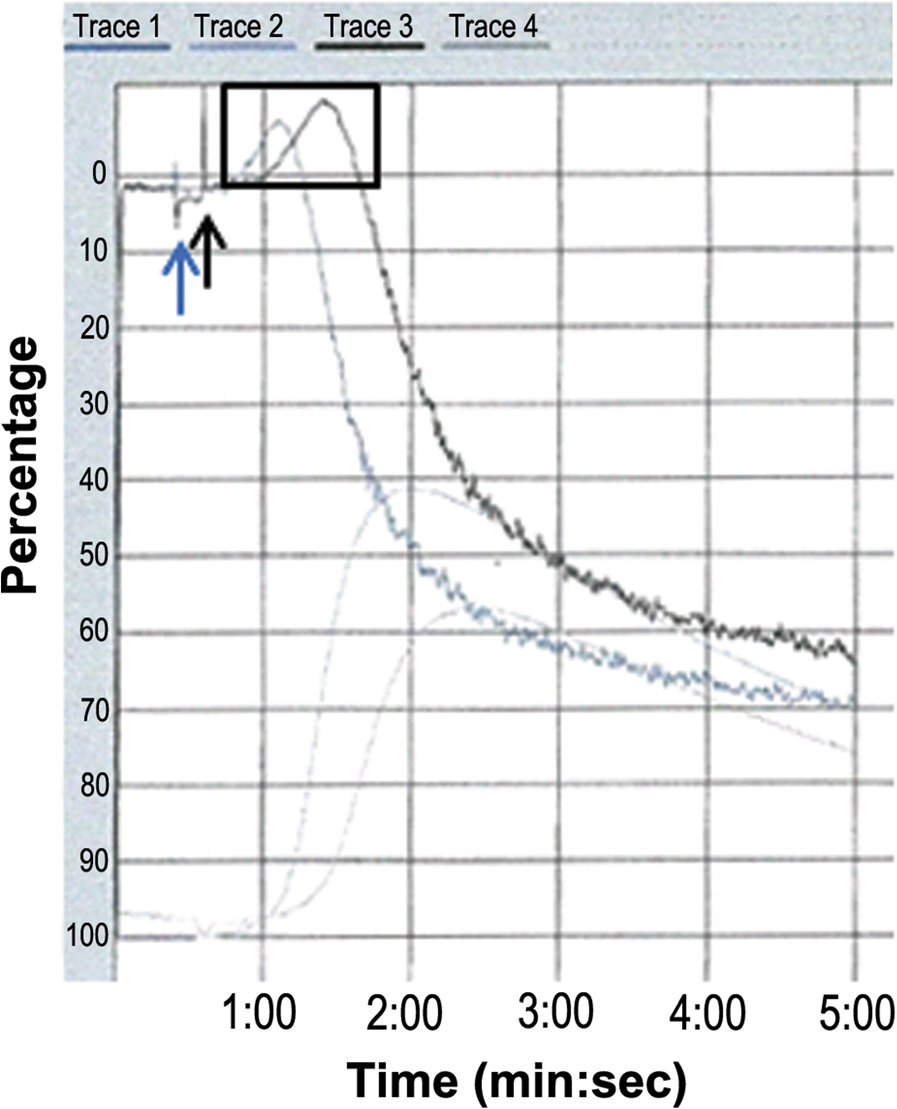
**More pronounced shape change of platelets of Nbea**^**+/-**^**mice upon collagen stimulation.** Collagen was added to wild-type and heterozygous platelet-rich plasma samples, respectively, at time points indicated by the blue and black arrows. Upon collagen stimulation, ATP secretion and aggregation were measured in the following 5 minutes. Trace 1 and 3 visualize the aggregation of platelets from Nbea^+/+^ and Nbea^+/-^ mice, respectively. The shape change preceding the aggregation is indicated with a black rectangle. All replication experiments show a similar, more pronounced shape change for Nbea^+/-^ mice. Traces 2 and 4 are the visualization of the ATP secretion of platelets of Nbea^+/+^ and Nbea^+/-^ mice, respectively (n = 4 mice/genotype). Quantification of total ATP secretion was done after corrections for background values (ATP secretion in platelet-poor plasma). Though it seems that there were differences between the ATP secretion curves for Nbea^+/+^ and Nbea^+/-^ mice in this experiment, quantification of ATP secretion values after correction for backgrounds in triplicate experiments, as shown in Table 1, showed no significant differences between genotypes.

Collagen binds to its glycoprotein receptors on the plasma membrane of platelets, resulting in the activation of phospholipase γ2 leading to an increase of intracellular Ca^2+^. The subsequent critical event is the reorganization of the actin cytoskeleton underlying filopodia and lamellopodia formation. This process is the essence of the platelet shape change which precedes platelet aggregation [38]. Although platelet aggregation after collagen stimulation was normal for Nbea^+/-^ mice (n = 4 mice/genotype; two-tailed *t*-test, *P* = 0.82) (Table 1), they consistently presented with a more pronounced shape change after collagen activation (Figure 2).

### Proteomic profile of platelets heterozygous for Nbea

To identify proteins differentially expressed in platelets of Nbea^+/-^ mice, a 2D-DiGE experiment was performed (n = 4 samples/genotype). A representative gel of the protein profile of platelets of Nbea^+/+^ and Nbea^+/-^ mice is shown in Additional file 1. Each gel contained at least 1,432 genuine protein spots, based on a manual verification of the three-dimensional profile characteristics. Only proteins present in at least 50% of the gel images were included for statistical analysis. A difference in expression was found for a total of 21 proteins, with 10 proteins having a reduced expression level and 11 proteins with an increased expression level in platelets of Nbea^+/-^ mice compared to platelets of Nbea^+/+^ mice (Additional file 1). Post-translational modifications probably explain the different positions of the identified proteins on the gel. However, no significant differences in expression level were observed, as the change in these 21 proteins was never greater than 1.3-fold, the reliable threshold for differential expression in 2D-DiGE experiments.

**Figure 3:**
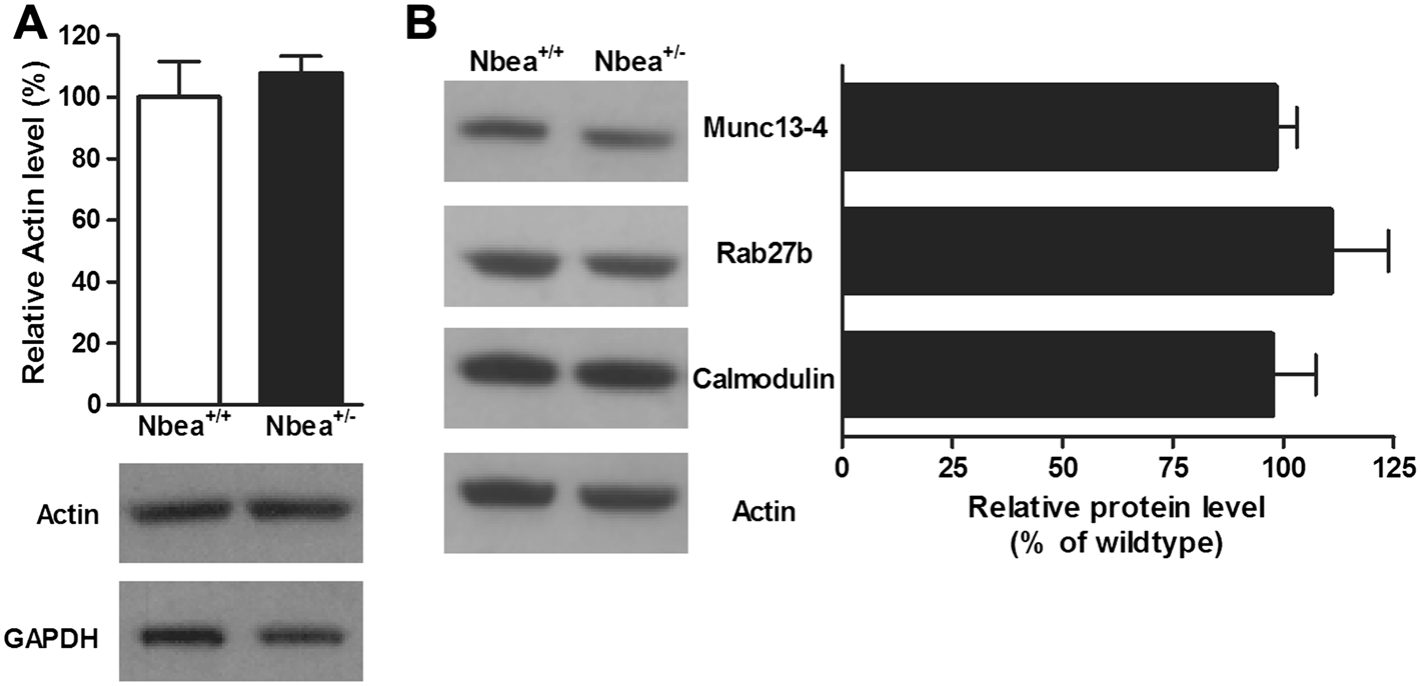
**Normal expression of actin and the proteins involved in dense granule formation and secretion in platelets of Nbea**^**+/-**^**mice. (A)** Western blot analysis detected no changes in the total actin levels in platelets of Nbea^+/-^ mice compared to Nbea^+/+^ mice. Actin expression was normalized to the glyceraldehyde-3-phosphate dehydrogenase (GAPDH) content and the expression in platelets of wild-type mice was set at 100% (n = 4/genotype). **(B)** The total amount of Munc13-4, Rab27b and Calmodulin was unaltered in platelets of Nbea^+/-^ mice. Protein levels were normalized to actin and the expression level in platelets of Nbea^+/+^ mice was set at 100% (n = 4 samples/genotype).

In addition, western blot was performed for several proteins related to dense granule biogenesis and secretion. The actin protein expression in platelets of Nbea^+/-^ mice was comparable with the actin levels in Nbea^+/+^ mice, although contradictory findings are reported with regard to altered total actin levels in Nbea^-/-^ mice [22,23,39,40]. Additional western blot analysis confirmed no differences in actin protein expression levels, when normalized to the GAPDH content, between Nbea^+/+^ and Nbea^+/-^ mice (n = 4 samples/genotype; two-tailed *t*-test, P = 0.56) (Figure 3A). Therefore, total actin level can be used as an internal control for western blot analysis. The expression of Munc13-4, Rab27b and Calmodulin after actin normalization did not significantly differ between Nbea^+/+^ and Nbea^+/-^ mice (n = 4 samples/genotype; two-tailed *t*-test; Munc13-4, *P* = 0.77; Rab27b, *P* = 0.51; Calmodulin, *P* = 0.81) (Figure 3B).

### Differential peptidomics of platelets of Nbea^+/-^ mice

Platelets contain a wide variety of peptides as well as proteins [41]. The peptide content of platelets of wild-type and heterozygous Nbea mice were compared by LC Q-TOF MS. Only peptides that were sufficiently abundant, well-aligned in time and present in at least three out of five samples were considered for statistical analysis. This resulted in approximately twenty differential peptides of which six could be identified (n = 5 mice/genotype) (Table 2). Quantification revealed significantly lower levels of six peptides, namely Thymosin β4 (full length and fragments containing amino acids (AA) 1 to 18 and 19 to 43; two-tailed *t*-test, *P* = 0.048), full length Thymosin β10 (two-tailed *t*-test, *P* = 0.020), Talin-1 (peptide containing AA 449 to 465; two-tailed *t-*test, *P* = 0.032) and the C-terminal part of Transgelin-2 (peptide containing AA 178 to 199; two-tailed *t*-test, *P* = 0.028). Interestingly, all these proteins are described as modulators of the actin cytoskeleton.

**Table 2:** **Differentially expressed peptides in platelets of Nbea**^**+/-**^**mice**

**Identification**	**Uniprot ID**	**MW (kDa)**	**Identified peptide**	**Mono-isotopic mass**	**Peptide sequence**	**Modification**	**Identification**	**WT/HET ratio**	***P***
Thymosin β4	P20065	5.05	TYB4	4960.44	SDKPDMAEIEKFDKSKLKKTE	Acetylation	MS/MS, clustering [42] and [41]	5.2433	0.048
					TQEKNPLPSKETIEEKQAGES				
			TYB4	2150.07	SDKPDMAEIEKFDKSKLK	Acetylation	Clustering [42] and [41]	3.9551	0.024
			(1–18)						
			TYB4	2828.36	KTETQEKNPLPSKETIEQEKQAGES		Clustering [42] and [41]	4.4780	0.036
			(19–43)						
Talin-1	P26039	269.8	TLN	1829.88	LPAIMRSGASGPENFQVG		Clustering [42] and identified in HEK MS/MS	2.4572	0.032
			(449–465)						
Transgelin-2	Q9WVA4	22.4	TAGLN2	2295.06	MGTNRGASQAGMTGYGMPRQIL		MS/MS and [41]	2.1505	0.028
			(178–199)						
Thymosin β10	Q6ZWY8	5.03	TYB10	4933.43	ADKPDMGEIASFDKAKLKKTE	Acetylation	MS/MS	2.0944	0.020
					TQEKNTLPTKETIQEKRSEIS				

The molecular weight (MW) is the MW of the precursor. A positive WT/HET ratio indicates a decrease in intensity in platelets of Nbea^+/-^ mice. The length of the identified peptide is indicated between brackets with the numbers corresponding to the respective amino acids. Genotype differences were tested using the two-tailed *t*-test (n = 5 mice/genotype).

### Altered cleavage of Talin-1 and altered phosphorylation of Calpain-2 in Nbea^+/-^ mice

**Figure 4:**
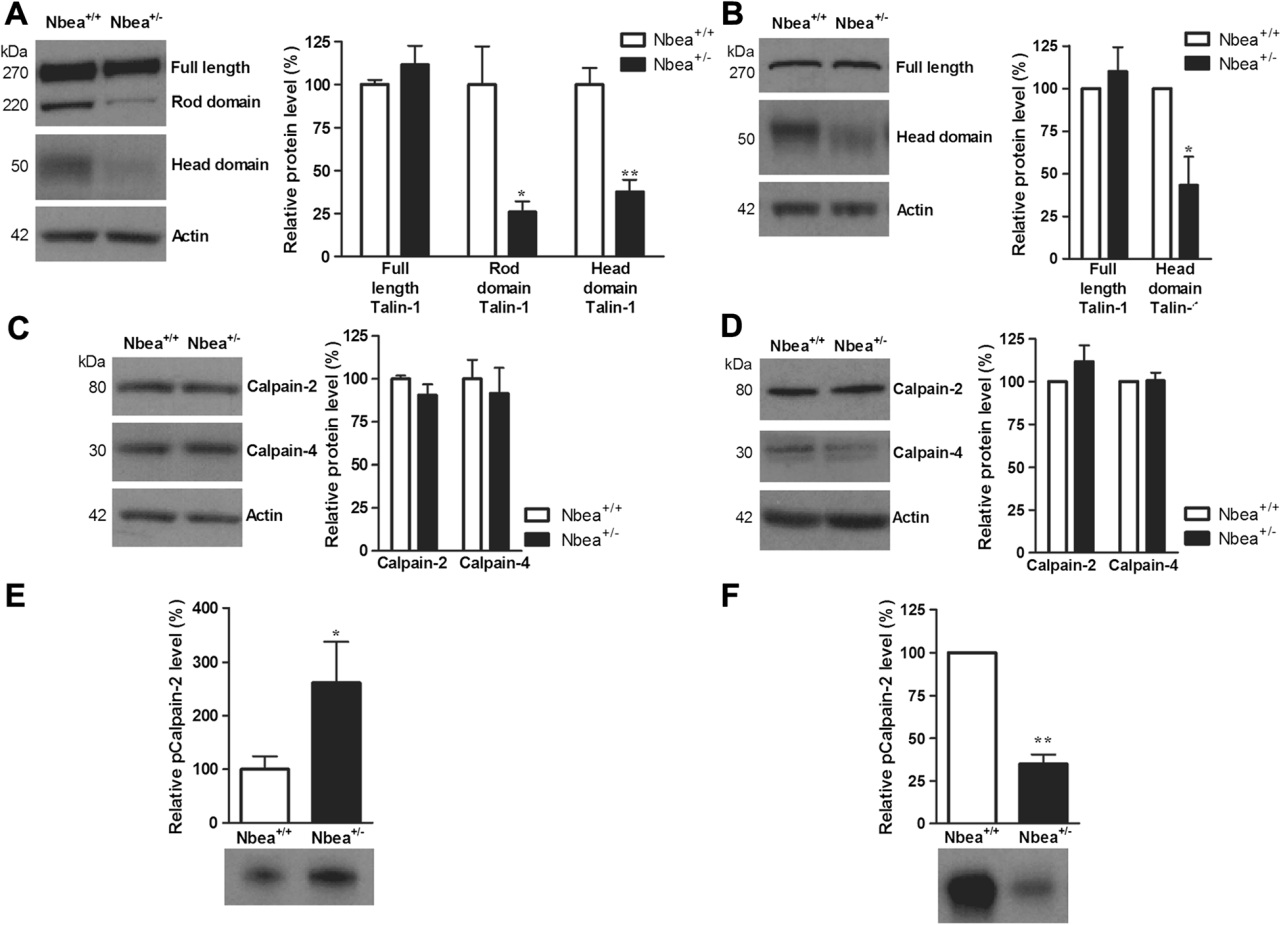
**Altered processing of Talin-1 and altered phosphorylation of Calpain-2. (A, B)** As well as the normal levels of full-length Talin-1, the presence of the cleavage products (head and/or rod domain) was significantly reduced in platelets **(A)** and in brain tissue **(B)** of Nbea^+/-^ mice (n = 4 samples/genotype). **(C**, **D)** Western blots of Calpain-2 and -4, responsible for Talin-1 cleavage, indicated a similar expression of both Calpain subunits in platelets **(C)** and in total brain lysates **(D)** of Nbea^+/-^ and Nbea^+/+^ mice (n = 4 samples/genotype and n = 3 samples/genotype, respectively). **(E)** Immunoprecipitation of phospho-(Ser/Thr) protein kinase A (PKA) substrates in platelets followed by Calpain-2 western blot revealed an increase in PKA-mediated phosphorylation of Calpain-2 (n = 6 samples/genotype). **(F)** Immunoprecipitation of phospho-(Ser/Thr) PKA substrates in total brain tissue followed by Calpain-2 western blot showed a decrease in PKA-mediated phosphorylation of Calpain-2 (n = 4 samples/genotype).

The peptide identified as Talin-1 (AA 449–465) is most likely a degradation product as Talin-1 is a high-molecular-weight protein of 270 kDa. Talin-1 consists of two domains, a head domain (49 kDa) and a rod domain (220 kDa) joined by a linker region containing a Calpain-2 (m-Calpain) cleavage site [43,44]. Western blot was performed for Talin-1 to assess the expression level and cleavage in platelets of Nbea^+/-^ mice (n = 4 samples/genotype). The expression level of full-length Talin-1 was slightly increased in platelets of Nbea^+/-^ mice but the difference was not significant (two-tailed *t*-test, *P* = 0.35) (Figure 4A). However, the presence of cleavage products of Talin-1 was markedly reduced in platelets of Nbea^+/-^ mice compared to Nbea^+/+^ mice, as the expression of both the rod and head domain was significantly decreased (two-tailed *t*-test, *P* = 0.018 and *P* = 0.002, respectively) (Figure 4A). To extrapolate these findings to brain, Talin-1 cleavage was studied in total brain lysates (n = 5 samples/genotype). As with platelet samples, the presence of the head domain was significantly reduced in the brain tissue of Nbea^+/-^ mice compared to Nbea^+/+^ mice (*t*-test for single means, *P* = 0.04) (Figure 4B) and full-length Talin-1 showed no significant difference in expression (*t*-test for single means, *P* = 0.55). There were several unsuccessful attempts to quantify the presence of the rod domain of Talin-1, due to a low signal-to-noise ratio for this domain. However, the reduced ratio of the head domain versus full-length Talin-1 indicates that in the brain tissue of Nbea^+/-^ mice the cleavage of Talin-1 is also reduced.

The protease responsible for the cleavage, Calpain-2, is a large catalytic subunit which forms a heterodimer with a regulatory subunit, Calpain-4 (Calpain small subunit 1) [45]. In order to investigate the reduced cleavage of Talin-1, western blot for Calpain-2 and -4 was performed and indicated similar expression levels of Calpain-2 and -4 in platelets (n = 4 samples/genotype; two-tailed *t*-test, *P* = 0.31 and *P* = 0.67, respectively) (Figure 4C) and in total brain lysates (n = 3 samples/genotype; *t*-test for single means, *P* = 0.34 and *P* = 0.92, respectively) (Figure 4D) of wild-type and heterozygous Nbea mice.

The activity of Calpain-2 is negatively correlated with its phosphorylation status at a PKA consensus site [46,47]. As Nbea is an AKAP protein, Nbea haploinsufficiency might result in defects in the sequestering of inactive PKA and compartmentalization of PKA, leading to altered PKA activity in different subcellular locations [48]. Therefore, the phosphorylation status of Calpain-2 in resting platelets and brain was examined. The lack of an anti-phosphoCalpain-2 antibody specific for PKA phosphorylation was overcome by performing immunoprecipitation with a phospho-(Ser/Thr) PKA substrate antibody detecting proteins containing a phosphorylated serine or threonine with an arginine at the -3 position, followed by western blot with Calpain-2 antibody. The level of phosphorylated Calpain-2 was significantly increased in platelets of Nbea^+/-^ mice with an average ratio of 2.8 (n = 6 samples/genotype; two-tailed *t*-test, *P* = 0.044) (Figure 4E). In contrast, the level of phosphorylated Calpain-2 in brain tissue of Nbea^+/-^ mice was significantly decreased by a factor of 2.86 (n = 4 samples/genotype; *t*-test for single means, *P* = 0.001) (Figure 4F).

**Figure 5:**
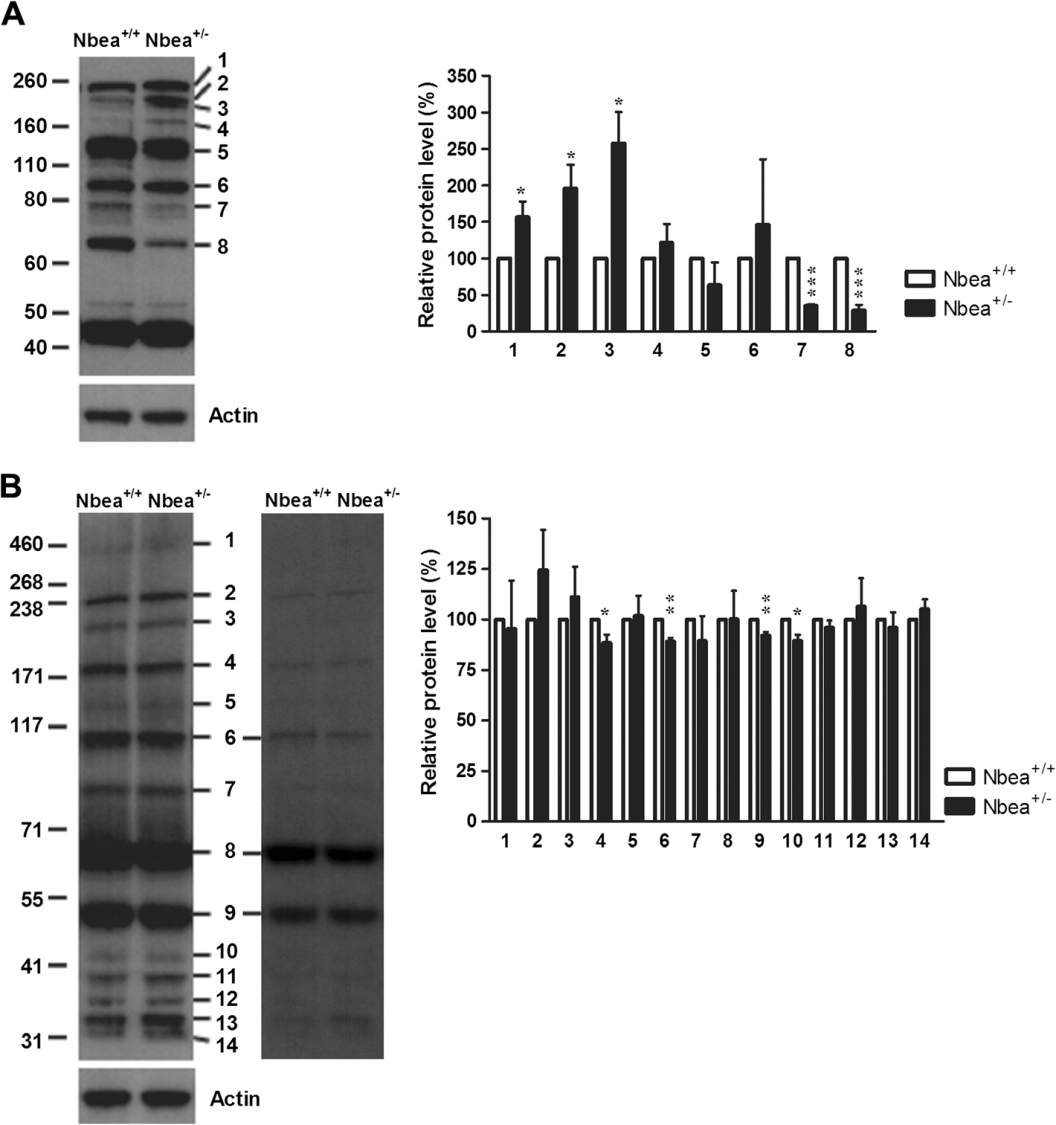
**Abnormal Protein kinase A*(*PKA) phosphorylation pattern in Nbea**^**+/-**^**mice.** Visualization of proteins phosphorylated by PKA revealed several changes in phosphorylation levels of proteins in resting platelets **(A)** and brain lysates **(B)** of Nbea^+/-^ mice. Phosphorylation levels were first calculated within a litter, followed by *t*-test for single means, with the mean of wild-type samples being 100%, to detect significant differences between Nbea^+/+^ and Nbea^+/-^ mice. **(A)** In resting platelets the three proteins with the highest molecular weight had a significantly higher phosphorylation status. The following three bands had an unaltered phosphorylation level whereas the last two bands had a significantly reduced phosphorylation profile (n = 4 samples/genotype). **(B)** Two different exposure times are shown for the PKA phosphorylation pattern of brain tissue, which were used for quantification. Image analysis revealed a significant decrease in phosphorylation level of four different proteins in brain tissue of Nbea^+/-^ mice (n = 5 samples/genotype).

To examine whether Nbea haploinsufficiency has a more general effect on PKA-dependent phosphorylation, western blot of platelet lysate with the phospho-(Ser/Thr) PKA substrate antibody was performed. Several phosphorylated proteins were detected in resting platelets of Nbea^+/+^ and Nbea^+/-^ mice and the phosphorylation of the three proteins with the highest molecular weight was significantly increased in Nbea^+/-^ mice (n = 4 samples/genotype; *t*-test for single means, band 1, *P* = 0.042; band 2, *P* = 0.027; band 3, *P* = 0.010) (Figure 5A). Additionally, the phosphorylation of two other proteins was significantly reduced (*t*-test for single means, band 7, *P* <0.001 and band 8, *P* <0.001) whereas the phosphorylation of proteins 4 to 6 was unaltered (*t*-test for single means, *P* = 0.44, *P* = 0.30 and *P* = 0.62, respectively) (Figure 5A). The same study was performed in brain tissue, which rendered several phosphorylated proteins. The phosphorylation of band 4, 6, 9 and 10 was significantly reduced in brain tissue of Nbea^+/-^ mice (n = 5 samples/genotype; *t*-test for single means, band 4, *P* = 0.047; band 6, *P* = 0.003; band 9, *P* = 0.009; band 10, *P* = 0.024) (Figure 5B).

## Discussion

In this study we have confirmed a causal link between Nbea haploinsufficiency and abnormal granule morphology, similar to our previous observation in ASD patients [24]. Dense granules of Nbea^+/-^ platelets were significantly smaller and showed a more pronounced shape change than Nbea^+/+^ platelets. However, none of the 1,432 proteins identified in a proteomic screen was differentially expressed. Differential peptidomics of platelets did identify four actin-interacting peptides with a reduced presence in platelets of Nbea^+/-^ mice. Validation of the peptidomic screen revealed reduced cleavage of Talin-1, most likely due to increased phosphorylation of Calpain-2 by PKA. The reduced cleavage of Talin-1 was confirmed in Nbea^+/-^ total brain, but phosphorylation of Calpain-2 was decreased. The importance of Nbea as a regulator of PKA activity was further substantiated, as haploinsufficiency of Nbea positively and negatively affects PKA-mediated phosphorylation of a multitude of proteins in resting platelets and in brain.

Normal serotonin levels were detected in platelets and serum of Nbea^+/-^ mice, which is consistent with the similar amount of dense granules per platelet in Nbea^+/+^ and Nbea^+/-^ mice as serotonin is taken up by platelets and stored in the dense granules [37]. Hyperserotonemia is one of the few biochemical anomalies reported in ASD patients and several possible causes have been suggested, such as increased synthesis or altered release of serotonin from enterochromaffin cells [49,50]. However, only 30% of ASD patients have been classified as being hyperserotonemic and increased levels of serotonin have also been detected in relatives of ASD patients [30,51]. These findings highlight the controversy regarding hyperserotonemia as a biomarker for ASD.

Several lines of evidence point to alterations in the cytoskeleton of platelets of Nbea^+/-^ mice. First, an increased shape change upon collagen stimulation was observed for platelets of Nbea^+/-^ mice. Interestingly, platelets from patients with a dense granule secretion defect also presented with a more pronounced shape change [31]. A similar shape change was noticed in platelets of patients with Duchenne muscular dystrophy, a disease characterized by a disturbed cytoskeletal organization [52]. Second, all peptides that have a significantly different level in platelets of Nbea^+/-^ mice are actin-interacting peptides. Transgelin-2, also named SMβ22, was identified as an actin-associated protein with an unknown function [53]. Thymosin β4 and β10 are the main intracellular G-actin sequestering peptides present in most mammalian cells. Binding of Thymosin β to G-actin prevents the polymerization to F-actin and reduced levels of Thymosin β4 and β10 lead to excessive formation of F-actin [54]. A decreased presence of Thymosin β4 and β10 in platelets of Nbea^+/-^ mice might be indicative for a decreased expression in neurons as well. This could contribute to the excessive F-actin clusters detected in the soma, dendritic shafts and axons of hippocampal neuronal cultures of Nbea^-/-^ mice and in the soma of cortical neurons of Nbea^+/-^ mice, although the exact mechanism remains to be determined [22]. Of note, a proteomic study using platelets from patients with a dense granule secretion defect also revealed actin-binding proteins as the major change in differentially expressed proteins compared to control platelets [31].

Multiple F-actin binding sites have been identified in Talin-1, both in the head and rod domain [55,56]. The peptide of Talin-1 identified in the peptidomic screen is likely to be a degradation product of the rod domain. The diminished presence of this peptide in platelets of Nbea^+/-^ mice correlates with the reduced presence of the rod domain detected on western blot. Talin-1 is cleaved by Calpain-2 after amino acid 432, yielding the head and rod domain of Talin-1 [44]. Calpain-2 activity is regulated by phosphorylation of PKA; more specifically, phosphorylation of Ser369 or Thr370 results in decreased activity [46,47]. The observed increase in PKA-specific phosphorylation of Calpain-2 is therefore a likely cause of the reduced cleavage of Talin-1 in blood platelets.

It is known that the F3 subdomain in the head domain of Talin-1 interacts with the cytoplasmic tail of β-integrin during the platelet activation process [57]. Therefore, alterations in platelet function might be expected based on the reduced presence of the head domain of Talin-1. However, in contrast to the conditional-knockout mouse models generated to study the molecular function of Talin-1, no complete ablation of Talin-1 was detected in platelets of Nbea^+/-^ mice. Of note is the finding that heterozygous conditional Talin-1-knockout mouse models do not present alterations in platelet function [58].

In Wistar rats, Talin is detected at the membranes of granules and dense compartments in the megakaryocytes [59,60]. Wistar Furth rats, a rat model with abnormal thrombocytopoietic phenotype associated with morphologically aberrant megakaryocytes lack these dense compartments. It is hypothesized that the lack of cytoskeletal proteins such as Talin may be responsible for the absence of the dense compartments in megakaryocytes of Wistar Furth rats. Therefore, it is possible that the observed alterations in Talin-1 in Nbea^+/-^ platelets contribute to the morphological differences of dense granules resulting in the reduced size of these dense granules.

Decreased cleavage of Talin-1 was also observed in total brain of Nbea^+/-^ mice. However, PKA-mediated phosphorylation of Calpain-2 was decreased, leading to increased activity, in contrast to blood platelets. A possible explanation for this discrepancy between platelets and brain tissue is that platelets represent a pure cell population, whereas the brain is a mixed-cell population. Therefore, it is possible that cells with reduced phosphorylation of Calpain-2 are not the cells with high Talin-1 expression. For instance, the brain tissue consists of both excitatory and inhibitory neurons and earlier studies using neuronal cultures derived from either adult Nbea^+/-^ mice or E18 Nbea^-/-^ mice revealed an imbalance in excitatory and inhibitory signaling with a more affected inhibitory neurotransmission [22,23,39]. Likewise, the phosphorylation status of Calpain-2 might be influenced by the excitatory/inhibitory imbalance present in the brain of Nbea^+/-^ mice. In addition, the expression level of Nbea varies in different brain regions [16], which is likely to contribute to the alterations in PKA mediated phosphorylation on Calpain-2 upon Nbea haploinsufficiency.

The altered level of PKA-phosphorylated Calpain-2 is in line with the reported increase in PKA-mediated phosphorylation of the cAMP response element-binding protein (CREB) in a neuroendocrine cell line after knockdown of Nbea, and indirectly, the increased level of brain-derived neurotrophic factor (BDNF), a target of phospho-CREB, in Nbea^+/-^ mice [40]. As well as Calpain-2, the phosphorylation status of other proteins is affected in Nbea^+/-^ mice, as indicated by the increased or decreased intensity of several proteins detected in a western blot of lysates of resting platelets or brain tissue with anti-phospho-(Ser/Thr) PKA substrate antibody. Although the alterations in PKA-mediated phosphorylation in total brain tissue from Nbea^+/-^ mice are more subtle, this might also be the result of the mixed-cell population and variable Nbea expression levels. The concurrence of increased, unaltered and decreased PKA phosphorylation of different proteins caused by Nbea haploinsufficiency can be explained by the compartmentalizing role of AKAPs. Because of its AKAP domain, Nbea belongs to the AKAP family of proteins, which is known to scaffold PKA near its target proteins in a distinct subcellular compartment. Different AKAPs lead to different PKA compartmentalizations [48]. Haploinsufficiency of Nbea will lead to an altered subcellular distribution of PKA, which will cause a higher, lower or unchanged PKA-mediated phosphorylation of target proteins, depending on their subcellular presence. With a continuously expanding list of ASD candidate genes, there is increasing interest in signaling pathways linking ASD genetics with biological functions, such as growth and neurite outgrowth of developing neurons and synaptic function [61,62]. There is ample genetic evidence for the involvement of AKAPs in the etiology of ASD, and proteins encoded by 10 ASD-linked AKAP genes were shown to functionally integrate signaling cascades within and between several biological functions [63]. Haploinsufficiency of *NBEA*, one of the 10 ASD-linked AKAP genes, is thereby suggested to affect multiple pathways, most likely via the pleiotropic effect of altered PKA activity.

## Conclusions

The characterization of platelets of Nbea^+/-^ mice provides evidence for a causal relationship between NBEA haploinsufficiency in the patient and abnormal platelet morphology. Furthermore, the impaired cleavage of Talin-1, increased phosphorylation of Calpain-2, and an altered PKA-related phosphorylation fingerprint, emphasizes the importance of the AKAP domain of Nbea for its function. Spatiotemporal control of PKA activity appears to be an important physiological function of Nbea. These findings highlight that alterations in the phosphorylation status of proteins might contribute to the pathogenesis of ASD in at least a subgroup of patients.

## Abbreviations

2D-DiGE: Two dimensional-differential gel electrophoresis; AA: Amino acid; AKAP: A-kinase anchoring protein; ASD: Autism spectrum disorder; BEACH: Beige and Chediak-Higashi; CREB: CAMP response element-binding protein; Cy: Carbocyanine; DDT: Dithiothreitol; ELISA: Enzyme-linked immunosorbent assay; GAPDH: Glyceraldehyde-3-phosphate dehydrogenase; LC: Liquid chromatography; LDCV: Large dense-core vesicle; MPV: Mean platelet volume; MS: Mass spectrometry; MWU: Mann–Whitney *U*; PBS: Phosphate-buffered saline; PKA: Protein kinase A; PRP: Platelet rich plasma; Q-TOF: Quadrupole time-of-flight; SEM: Standard error of the mean; SNP: Single nucleotide polymorphism; SV: Synaptic vesicle

## Competing interests

The authors have no competing interests to declare.

## Authors’ contributions

KN and KT conducted experiments, analyzed and interpreted the data and drafted the manuscript. KB and EW conducted and analyzed experiments. MDM conducted experiments and assisted in the interpretation of the data. KF designed and conducted experiments, interpreted the data and helped to draft the manuscript. JC designed experiments, interpreted the data and helped to draft the manuscript. All authors read and approved the final manuscript.

## Supplementary Material

Additional file 1: Figure S1 The protein content of Nbea^+/-^ mice contained 21 differentially expressed proteins compared to Nbea^+/+^ mice, A representative gel of the protein profile of platelets of Nbea^+/+^ and Nbea^+/-^ mice after two dimensional-differential gel electrophoresis (2D-DiGE) is shown. Click here for file
