# Supplementary material for: Platelets of mice heterozygous for neurobeachin, a candidate gene for autism spectrum disorder, display protein changes related to aberrant protein kinase A activity
Source: Mol Autism. 2013 Nov 4;4:43. doi: 10.1186/2040-2392-4-43 (PMC3829668; doi:10.1186/2040-2392-4-43)
Supplement: Additional file 1: Figure S1 — The protein content of Nbea+/- mice contained 21 differentially expressed proteins compared to Nbea+/+ mice, A representative gel of the protein profile of platelets of Nbea+/+ and Nbea+/- mice after two dimensional-differential gel electrophoresis (2D-DiGE) is shown. [file 2040-2392-4-43-S1.docx]

Additional file 1


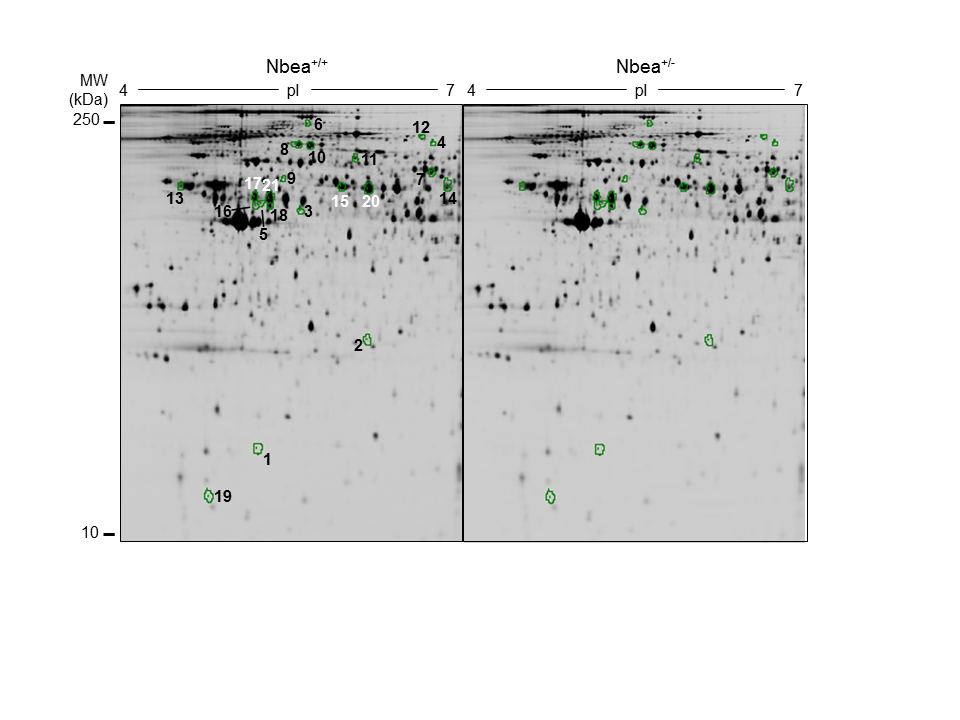


Figure S1: The protein content of Nbea^+/-^ mice contained 21 differentially expressed proteins compared to Nbea^+/+^ mice

In total, 21 proteins were differentially expressed in platelets of Nbea^+/-^ mice, marked on the gel with green surroundings and numbered (listed in Additional file 2). Numbers indicated in white relate to the identified proteins. (n = 4 samples/genotype) MW: molecular weight, pI: isoelectric point
